# Agitation and Sugar Craving Related to Epilepsy Seizure

**DOI:** 10.1155/2021/9969854

**Published:** 2021-05-03

**Authors:** Rania Adel Hameed, Mohammad Reza F. Aghdam

**Affiliations:** ^1^Department of Child and Adolescent Psychiatry, Innlandet Hospital Trust, Gjøvik, Norway; ^2^Landmo Nursing Home and Rehabilitation Center, Nordre Land, Norway

## Abstract

**Introduction:**

Epilepsy is a chronic central nervous system disorder characterized by the recurrence of unprovoked seizures and can affect people of all ages. Seizure symptoms can vary widely in patients. Many papers have been published about agitation and epileptic seizures, but almost nothing about sugar cravings and agitation related to epilepsy. The purpose of this case report is to shed light on possibly a hidden symptom within the epilepsy field, in fact sugar cravings. *Case presentation*. A 12-year-old boy was referred to the children and adolescent psychiatric outpatient clinic with suspicion of ADHD. The boy has struggled with anxiety, concentration, and impulsivity. Because of intense agitation and sugar cravings, the patient was referred to EEG. The EEG shows pathological activity with bilatero-temporal to central epileptiform activity, not synchronized. After pathological EEG findings, the patient started treatment with Lamotrigine. Great improvement when it comes to agitation, moodiness, and reduction of sugar craving after starting with Lamotrigine.

**Conclusion:**

We consider inexplicable behavior or symptoms such as agitation and sugar craving may be related to epilepsy seizure. Therefore, it is important that these patients should be examined more closely with EEG to confirm or deny epilepsy.

## 1. Introduction

Epilepsy is a chronic central nervous system disorder characterized by the recurrence of unprovoked seizures and can affect people of all ages [1–3]. Not all unprovoked seizures are due to epilepsy; drug-induced seizures and febrile seizures are such an example [4, 5]. Over 70 million people worldwide are affected by this disease. Epilepsy is a symptom complex with a strong genetic predisposition and multiple risk factors [6, 7]. Seizure symptoms can vary widely in patients. A relatively rare but serious comorbidity in epilepsy is represented by psychiatric disorders [8, 9]. The diagnosis is made with at least two unprovoked seizures plus a pathological EEG [10]. The classification of etiologies of epilepsies is divided into four main categories: idiopathic epilepsy, symptomatic epilepsy, provoked epilepsy, and cryptogenic epilepsy [11]. Epilepsy seizures itself classifies into four main groups: focal, generalized, combined generalized and focal, and unknown seizures based on abnormal brain activity [12, 13]. Agitation, irritability, and aggression are seen in a minority of people with epilepsy [14–16].

## 2. Case Presentation

A 12-year-old boy was referred to the children and adolescent psychiatric outpatient clinic with suspicion of ADHD. It is the third time reference with the same suspicion. First time reference was in 2011 and second time was 2014 where he did not meet the criteria for ADHD diagnosis. The third reference to the children and adolescent psychiatric outpatient clinic was in 2018. The reference from the patient's family doctor was described concentration difficulties, difficulties with emotion regulation, impulse control, and regulation of activity and sleep. The patient met the criteria for the diagnosis of ADHD at third control.

### 2.1. Background

The patient was placed in a foster home when he was 8 months old. When he moved to his foster family, he was malnourished, dehydrated, and slept 18 hours a day. Based on the previous examination in the outpatient clinic, it appears that the boy has struggled with anxiety, concentration, and impulsivity since then. Some of his symptoms lead that the patient getting tired quickly and may fall asleep suddenly at school or in activity. At a younger age, he could suddenly fall asleep while sitting and playing in the sandbox. When he gets tired, he often gets headaches and visual disturbances. The last mentioned can disappear after getting some rest. His impulsivity shows by suddenly pushing a child he accidentally passes. He has difficulties to perceive messages and follow instructions. He strives to regulate the emotions, and the emotions fluctuate quickly and become unusually intense. When he gets angry, he gets very angry and apparently loses control of what he does. The patient was previously examined for sleep disorders. No suspicion of hypersomnia such as Kleine-Levin or narcolepsy was detected based on history taking (anamnesis). The patient had no signs or symptoms that could be related to obstructive sleep apnea syndrome (OSAS) either. He had problems with insomnia and poor sleep quality, no hypersomnia, snoring, or constant fatigue during the day.

During the consultation at the outpatient clinic, we did the following tests: Ability Test (WISC-IV), Kiddie SADS, Playing observation, BRIEF, and Barkley. A summary of these tests is shown in Table 1. It was taken a complete blood sample of the patient as a part of routine examination which was normal. The summary of laboratory test results is shown in Table 2.

General physical examination is the following: normal findings of the cardiac, pulmonary, and abdominal examination. Diadochokinesis test is the following: jerky and slow movements on both sides and scores 5 points. Finger opposition test is the following: slow with slightly uneven left-sided mirror movements and scores 5 points. Walk on the lateral side of the foot is the following: relaxed extremity bilaterally and scores 2 points. Total score is 12 points (6 points more than 15 percentile). Otherwise, normal findings of the neurological examination are examined.

**Figure 1:**
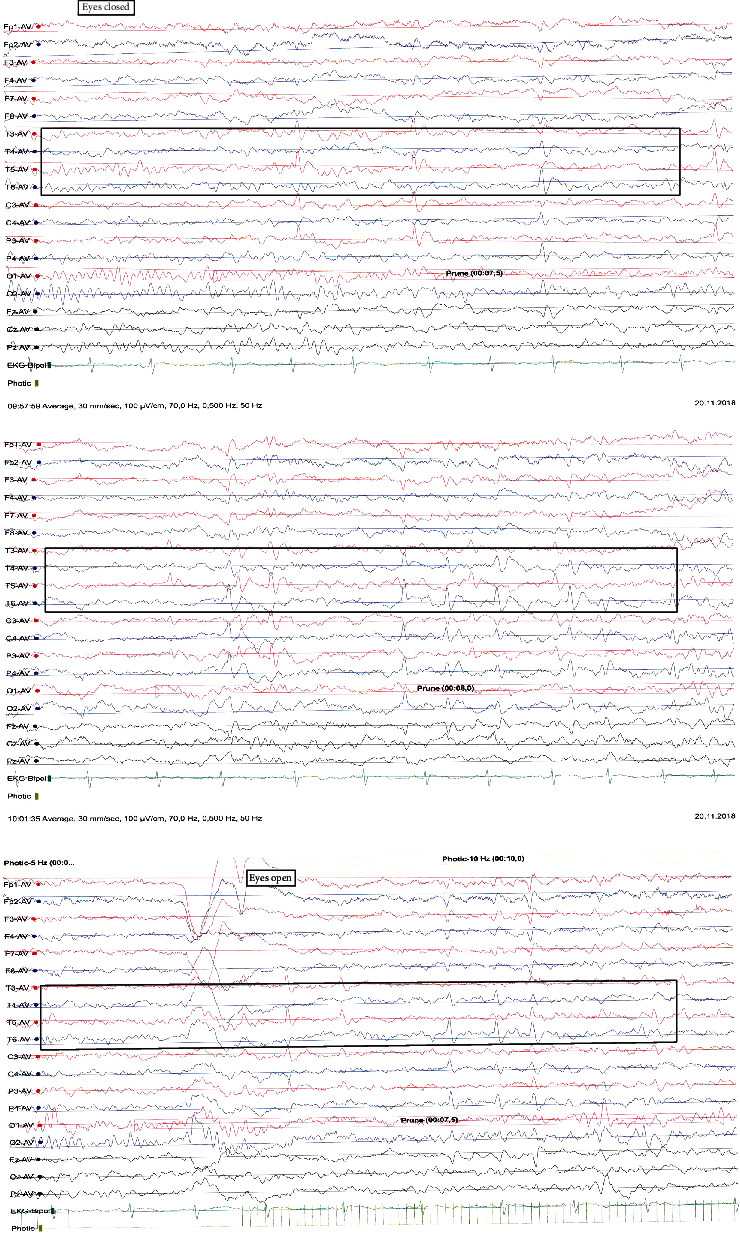
The EEG shows pathological activity with bilatero-temporal to central epileptiform activity, not synchronized.

Because of intense agitation and sugar cravings, the patient was referred to EEG. Generally, minor neurological abnormalities can be observed in children with, for example, hyperkinetic disorders/learning difficulties, or cognitive dysfunctions. The EEG from November 2018 shows pathological activity with bilatero-temporal to central epileptiform activity, not synchronized (Figure 1). There was no information about seizures, sudden uncontrolled twitching, or observations of collapse or fall down. The patient cannot remember things after the incident.

After pathological EEG findings, the patient started treatment with Lamotrigine. Great improvement when it comes to agitation, moodiness, and reduction of sugar craving after starting with Lamotrigine. However, concentration difficulties and hyperactivity are unchanged; this may be due to ADHD. CNS stimulants are scheduled to start soon.

## 3. Discussion

We present a case of a 12-year-old boy who was referred to the children and adolescent psychiatric outpatient clinic with suspected ADHD. The patient was referred to EEG, with suspicion of possible epilepsy based on intense agitation and sugar cravings. The suspicion of epilepsy was confirmed by pathological EEG findings. A number of articles have been published about aggression, agitation, and irritability in some patients with epilepsy [17, 18], but no clear sugar craving behavior is a possible symptom of epilepsy. In this case, it is difficult to draw any clear conclusion that sugar craving behavior may be a symptom related to epilepsy, since the patient has had both symptoms of agitation and sugar craving. Anyway, we could not ignore so clear symptom or behavior such sugar craving. Our suspicion of epilepsy related to agitation and sugar craving is particularly strengthened when the patient responds well to antiepileptic therapy (Lamotrigine). We are still uncertain about the cause or mechanism behind it. Epilepsy carries a greater risk of mortality than expected relative to the general population [19]. This makes the early and correct diagnosis of epilepsy important. Sometimes, it can be challenging to think about epilepsy especially when the patient does not have clear epilepsy signs or symptoms. We believe this case report contains a worthwhile clinical lesson for all health professionals, especially physicians. Physicians should keep this possibility in mind if a patient has unexplained behavior or symptom; it may be related to an atypical or hidden epilepsy.

## 4. Conclusion

We consider inexplicable behavior or symptoms such as agitation and sugar craving may be related to epilepsy seizure. Therefore, it is important that these patients should be examined more closely with EEG to confirm or deny epilepsy.

## Figures and Tables

**Table 1:** Summary of the psychological tests.

Tests	Results/conclusion
Ability test (WISC-IV)	Intellectual abilities below the normal range of age, suggesting a need for school facilitation.
	Low scores on verbal comprehension index. Sprawling result between the subtests.
	Numerous scores may sometimes indicate that scores have been affected by conditions that the test is not initially intended to measure (e.g., such error sources may be specific learning difficulties, concentration, or daily form). During testing, the patient had good concentration is observed during the first minutes but decreasing concentration and increasing motor turbulence beyond testing.

Kiddie SADS	Meets 8/9 criteria for attention deficit and 6/9 criteria for hyperactivity/impulsivity.
	The symptoms have lasted longer than 6 months. And was present before the age of seven.
	Impaired function appears in several areas. Meets 6/8 criteria for oppositional defiant
	Disorder (ODD). The symptoms cause significant social impairment. The symptoms have lasted for more than 6 months. The criteria for tics may be met (either chronic vocal or transient tics).

Playing observation	Play observation was conducted in two hours in the activity room. He makes good contact with therapists and uses eye contact, mimicry, and smiles in a way that is perceived as mutually and socially regulative. He pretends to understand the nonverbal signals during the Mikado-game. He shares joy with his therapist by smiling while he playing ping-pong.
	He seems to concentrate well on activities along with therapist during playing billiards, ping-pong, and chess. There are no signs of inattention in these situations. A high level of activity is observed through the hours. When there are breaks, he is tricking, for example, tricking with his milk carton. Otherwise, no clear signs of impulsivity are observed during activities.

BRIEF	Clinical scales are based on the following area: impulse inhibition, emotional control, initiation, working memory, planning/organizing, and monitoring. Father fills out the form: all of the aforementioned scales are in clinical area. Mother fills out the form: of the overall scales, behavioral regulation and global executive function were in the clinical area, while metacognition was on the border. Teacher fills out the form: the overall scales were metacognition and global executive function in clinical area, while the behavioral regulation was below but close to the clinical area.
	Assessment/evaluation: the results of the brief test from home and school indicate executive difficulties.

Barkley	Father fills out the form and the patient scores over 98 percentile on inattention, hyperactivity, and impulsivity. Mother fills out the form and the patient scores over 95 percentile on inattention, hyperactivity, and impulsivity. Teacher fills out the form and the patient scores over 93 percentile on inattention, hyperactivity, and impulsivity. Assessment/evaluation: the results of the Barkley test from home indicate difficulties with attention, hyperactivity, and impulsivity. However, the results of the Barkley test from school are below the clinical threshold.

**Table 2:** Summary of laboratory test results.

Test	Result	Unit	Reference range
Hemoglobin	13.5	g/dL	11,0–14, 0
Hematocrit	0.40	L/L	0,32–0,44
Erythrocytes	4.5	x10E12/L	4,0–5,2
Leukocytes	4.5	x10E9/L	4,5–14
Platelets	313	x10E9/L	145–390
Neutrophils	2	x10E9/L	2,0–8,0
Lymphocyte	2.1	x10E9/L	1,5–6,0
Monocytes	0.3	x10E9/L	0,2–1,0
Eosinophils	0.1	x10E9/L	0,1–0,4
Basophils	0	x10E9/L	0,0–0,1
Sodium	141	mmol/L	137–145
Potassium	4.8	mmol/L	3,6–4,6
Calcium	2.54	mmol/L	2,15–2,51
25-OH vitamin D	78	nmol/L	50–150
Glucose	4.1	mmol/L	4,0–6,0
Creatinine	46	*μ*mol/L	30–60
AST	34	U/L	15–45
ALT	10	U/L	10–70
IgA	2.4	g/L	0,4–3,3
TSH	2.7	mIE/L	0,27–4,20
T4	13	pmol/L	12,0–22,0
IgE total	21	kU/l	0–148
IgE food panel fx5	<0,10	kU/l	0–0,34
Antitissue transglutaminase (IgA)	<7	U/ml	0–7
Celiac disease investigation	Negative		Negative
